# The influence of interviewers on survey responses among female sex workers in Zambia

**DOI:** 10.1186/s12874-019-0703-2

**Published:** 2019-03-15

**Authors:** Guy Harling, Michael M. Chanda, Katrina F. Ortblad, Magdalene Mwale, Steven Chongo, Catherine Kanchele, Nyambe Kamungoma, Leah G. Barresi, Till Bärnighausen, Catherine E. Oldenburg

**Affiliations:** 1 Institute for Global Health, University College London, London, UK; 2 Department of Global Health, University of Washington, Seattle, WA USA; 3 Department of Epidemiology, Harvard T.H. Chan School of Public Health, Boston, MA USA; 4 Africa Health Research Institute, KwaZulu-Natal, South Africa; 5 John Snow, Inc, Lusaka, Zambia; 6 Heidelberg Institute of Global Health, Faculty of Medicine, University of Heidelberg, Heidelberg, Germany; 7 Francis I. Proctor Foundation, University of California, San Francisco, CA USA; 8 Department of Ophthalmology, University of California, San Francisco, CA USA; 9 Department of Epidemiology and Biostatistics, University of California, San Francisco, CA USA

**Keywords:** Gender, Interviewer, Validity, Zambia, Female sex workers, Gender-based violence

## Abstract

**Background:**

Interviewers can substantially affect self-reported data. This may be due to random variation in interviewers’ ability to put respondents at ease or in how they frame questions. It may also be due to systematic differences such as social distance between interviewer and respondent (e.g., by age, gender, ethnicity) or different perceptions of what interviewers consider socially desirable responses. Exploration of such variation is limited, especially in stigmatized populations.

**Methods:**

We analyzed data from a randomized controlled trial of HIV self-testing amongst 965 female sex workers (FSWs) in Zambian towns. In the trial, 16 interviewers were randomly assigned to respondents. We used hierarchical regression models to examine how interviewers may both affect responses on more and less sensitive topics, and confound associations between key risk factors and HIV self-test use.

**Results:**

Model variance (ICC) at the interviewer level was over 15% for most topics. ICC was lower for socio-demographic and cognitively simple questions, and highest for sexual behaviour, substance use, violence and psychosocial wellbeing questions. Respondents reported significantly lower socioeconomic status and more sex-work related violence to female interviewers. Not accounting for interviewer identity in regressions predicting HIV self-test behaviour led to coefficients moving from non-significant to significant.

**Conclusions:**

We found substantial interviewer-level effects for prevalence and associational outcomes among Zambian FSWs, particularly for sensitive questions. Our findings highlight the importance of careful training and response monitoring to minimize inter-interviewer variation, of considering social distance when selecting interviewers and of evaluating whether interviewers are driving key findings in self-reported data.

**Trial registration:**

clinicaltrials.gov NCT02827240. Registered 11 July 2016.

**Electronic supplementary material:**

The online version of this article (10.1186/s12874-019-0703-2) contains supplementary material, which is available to authorized users.

## Background

A substantial literature highlights that interviewers can affect survey responses at three levels: (i) unit non-response, i.e., declining to interview; (ii) item non-response, i.e., declining to answer a specific question; and (iii) item quality, i.e., not providing the true answer [1]. Most research to date has been on item quality, although there is some evidence that item non-response may also be affected by interviewers. Variation in responses across interviewers may reflect random differences in interviewers’ manner (e.g., how they frame or explain questions) and ability to draw out responses (e.g., how judgemental they seem). This may be controlled for through the use of hierarchical models accounting for interviewer-level variation [2].

Interviewers may also generate different response patterns due to systematic variation in their characteristics. Past research has highlighted many candidate characteristics, including gender, age, race/ethnicity, socioeconomic status (SES), research experience and personality [3]. Several theories posit how characteristics of the interviewer alone, or of the interviewer-respondent dyad, may affect responses. First, *social distance* theory suggests that when interviewers and respondents are similar, response rates and item quality should be higher, due to respondents being more at ease and more likely to be honest [4]. Second, *social desirability* theory suggests that respondents are likely to match their responses to what they believe the interviewer believes or wants to hear [5]. Finally, *social role* theory suggests that interviewer effects may be different for different types of question, with a particularly strong effect when asking about topics linked to roles expected to be espoused by interviewers, e.g., reporting more caring behaviour to female interviewers, reporting less racism to ethnic minority interviewers [6].

These theories can be illustrated with the example of gender [7]. Social distance theory predicts that responses will be more accurate for same-gender pairings for both male and female respondents. Social desirability theory in contrast predicts responses will vary by interviewer gender alone [8]. If both theories apply, we would expect to see an interaction of interviewer and respondent genders to generate four levels of response (male-male, male-female, female-male and female-female). Finally, social role theory predicts that differences between male and female interviewers would be greatest for those questions with the strongest gender expectations, e.g., greater reporting of caring behaviour to female interviewers.

Empirically, female interviewers appear to be considered more sympathetic, less judgmental and less threatening for a broad range of interview types [3, 9–11]. There is also evidence that same-gender interviewers elicit more responses, in particular to sensitive questions; i.e., those questions on which respondents believe they are most likely to be judged for their response [7, 12, 13]. Perhaps as a result, most studies find that female interviewers elicit more responses from female respondents, although the literature on male-male interviews is more mixed [3, 11, 14, 15].

Systematic interviewer variation in response for self-reported surveys has long been recognized for public health outcomes [12]. Interviewer gender is frequently considered, particularly for sexual behaviour questions, with a wide range of response patterns seen. These include an increased willingness for men to report sexual behaviours to women [14], for everyone to report sexual behaviours to same-sex interviewers [16] and for male military personnel in the Dominican Republic to report more sexual activity, but less alcohol use and sexual coercion, to female interviewers [17].

Within sub-Saharan Africa, findings on interviewer impact for sexual behaviour questions are also mixed, again largely focused on gender. One Ghanaian study found that men did not report differentially by interviewer gender; but women reported more prior sexual activity and concern about AIDS to male interviewers, and more often that condoms spoil sex to females [18]. In contrast, a study in South Africa found no effects for female respondents, but that men reported more sexual partners to female interviewers, and lower-risk behaviours to older interviewers [19]. A smaller Ghanaian cross-over study (respondents talking with both male and female interviewers) found no significant results [20].

The impact of interviewer characteristics has also been considered for gender-based violence (GBV) and intimate partner violence (IPV) questions [21]. Violence prevalence may be underreported due to reticence on the part of the interviewer or respondent to discuss the topic, due to low privacy, expected social roles or distress generated [22, 23]. Since some of these mechanisms may be gendered, some studies have adjusted for interviewer effects when measuring IPV [24, 25]. Explicit evaluation of gender-of-interviewer effects is limited, although a race-of-interviewer study for Africa-American respondents in the USA found little impact on IPV disclosure [26].

While interviewer effects have been examined in Africa, we are not aware of any work considering highly stigmatized populations, particularly when asking about potentially stigmatizing behaviours. However, these may be exactly the respondent populations and topics most likely to craft responses to fit narratives that either they have about themselves, or that they believe interviewers to have about them. We therefore analysed how the identities and characteristics of interviewers affected both risk factor prevalence and measures of association between variables in a survey of sexual and other experiences amongst female sex workers (FSWs) in three Zambian transit towns.

## Methods

We used data from the Zambian Peer Educators for HIV Self-Testing (ZEST) study, a cluster randomized trial of the impact of HIV self-testing provision among FSWs in Chirundu, Livingstone and Kapiri Mposhi [27, 28]. Peer educators, who were current or former FSWs, were recruited from existing female sex worker organizations operating in the study towns. Each peer educator recruited six women into the trial. Eligibility criteria were: (i) primarily living in one of the towns; (ii) being at least 18 years old; (iii) reporting exchanging sex for money, goods or other items of value at least once in the prior month; (iv) self-reporting either being HIV negative or of unknown serostatus; and (v) not having tested for HIV in the past 3 months. Peer educators referred potential participants from within their social networks to study staff who screened them for eligibility first by phone and then in-person by study staff. Respondents received 50 Zambian Kwacha (ZMW; ~US$5) per interview they completed and no incentive for participation in peer educator sessions; peer-educators were paid for their participation [27]. The study was reviewed by the Institutional Review Boards at the Harvard T.H. Chan School of Public Health in Boston, USA and ERES Converge in Lusaka, Zambia. Written informed consent was obtained from all participants.

The baseline survey lasted an average of 35 min. Each survey was conducted by a research assistant recruited locally, in the local language chosen by the respondent. Data were collected through a face-to-face, computer-assisted personal interview (CAPI) at a private and convenient location, using a tablet computer and the CommCare (Dimagi Inc., Cambridge, MA) electronic data capture platform. There were follow-up interviews at one and 4 months post-baseline.

Research assistants were hired in each town. Desirable qualifications included substantial education (preferably including some tertiary attendance), computer literacy and experience of working with FSWs. Many of those hired had past experiences working with FSWs through the Corridors of Hope project [29]. Assignment of research assistant interviewers to respondents was random at the level of the peer educator, within each town, and this assignment of peer educators to research assistants was made prior to study commencement.

We considered 80 variables captured in the baseline survey, ranging from non-sensitive to highly sensitive questions, in four overarching categories: (i) socio-demographics; (ii) sex work; (iii) sexual behaviour and health; and (iv) other HIV risk factors – including history of abuse, substance use, interactions with law enforcement and psychosocial wellbeing (depression, HIV stigma, social support and self-efficacy). Tables 1, 2, 3 and 4 contain a detailed list of variables. We also considered self-reported testing for HIV since baseline at one-month follow-up, and testing in the past month at four-month follow-up.

**Table 1:** Socio-demographic responses by ZEST study population

	Univariate		Bivariate regression ^b^			
	N	% / Median [IQR]	ICC^c^	Male IVR	Female IVR	*p*-value
Study site	965					
Livingstone		49.7%				
Kapiri		25.4%				
Chirundu		24.9%				
Age	965	25 [21–30]	3.9%	26.2	26.3	0.99
Ever married	965	69.9%	12.5%	68.7%	75.5%	0.69
Currently divorced/separated	965	24.5%	10.4%	26.8%	18.4%	0.55
Education	965					0.65
No formal education		11.2%		7.3%	11.8%	
Primary (up to 9 years)		46.6%		44.6%	53.0%	
> 9 years		42.2%		48.1%	35.2%	
Able to read and write	959	75.3%	9.1%	82.8%	67.7%	0.06
Monthly income (ZMW)	949					0.38
No income		21.3%		2.6%	24.8%	
< 250 kwacha		13.0%		9.9%	38.4%	
251–500 kwacha		24.8%		39.1%	29.6%	
501–1000 kwacha		25.9%		37.4%	6.2%	
1001–1500 kwacha		7.7%		6.9%	0.6%	
> 1500 kwacha		7.4%		4.2%	0.4%	
Financial situation	962					**0.020**
Very poor		14.7%		4.8%	24.1%	
Poor		37.8%		32.8%	54.1%	
Just getting by		36.3%		46.5%	18.9%	
Comfortable		10.0%		14.2%	2.6%	
Very comfortable		1.2%		1.8%	0.3%	
Mobile phone ownership	965	85.0%	6.3%	86.4%	85.2%	0.94
Self-perceived relative SES ^a^	965	3 [2–5]		3.1	3.0	0.94
Any income from non-sex-work	965	30.2%	7.5%	30.6%	27.3%	0.87

*ICC* Intraclass Correlation Coefficient, *IQR* inter-quartile range, *IVR* interviewer, *SES* socio-economic status, *ZMW* Zambian Kwacha: 1 Kwacha ~US$ 10

^a^10-point scale

^b^All bivariate regressions included study site fixed effects and interviewer random intercepts. Values for male and female IVR are marginal predicted values based on regression coefficients. ^c^ ICC is the proportion of all variance in a model without interviewer gender attributable to variation in interviewer identity; not available for Poisson or ordered logistic models. *P*-value is for a *χ*^2^ test, adjusted for multiple testing across all results shown in Tables 1, 2, 3 and 4 using the Benjamini-Hochberg method. *P*-values <0.05 shown in bold

**Table 2:** Sex work responses by ZEST study population

	Univariate		Bivariate regression ^a^			
	N	% / Median [IQR]	ICC ^b^	Male IVR	Female IVR	*p*-value
Age at first sex for money	949	19 [17–21]	1.6%	19.8	19.0	0.38
Condoms available while working	963	1.8%				0.26
Never		1.8%		1.2%	2.1%	
Seldom		4.6%		3.2%	5.3%	
Sometimes		62.7%		60.7%	68.3%	
Often		8.3%		9.7%	7.7%	
Always		22.6%		25.1%	16.6%	
Ask SWC to use a condom	963					0.55
Never		3.8%		2.2%	4.0%	
Seldom		5.9%		3.7%	6.4%	
Sometimes		54.2%		53.2%	62.2%	
Often		11.7%		14.5%	11.0%	
Always		24.3%		26.5%	16.4%	
SWCs ask to use a condom	965					0.49
Never		15.3%		16.2%	7.8%	
Seldom		18.2%		24.7%	15.8%	
Sometimes		57.7%		54.7%	66.7%	
Often		5.0%		2.6%	5.6%	
Always		3.7%		1.8%	4.1%	
SWCs request that not to use a condom	965					0.46
Never		10.1%		10.3%	4.6%	
Seldom		13.8%		19.1%	10.9%	
Sometimes		51.5%		55.4%	54.5%	
Often		19.5%		12.7%	24.1%	
Always		5.2%		2.4%	5.8%	
ZMW price for vaginal sex with condom	931	100 [50–150]	6.6%	101	99	0.95
ZMW price for vaginal sex without condom	883	150 [100–250]	7.0%	203	177	0.58
ZMW price for anal sex with condom	220	150 [70–200]	16.7%	146	185	0.65
ZMW price for anal sex without condom	213	200 [130–300]	14.8%	242	263	0.89
Average nightly # of SWCs: other FSW	954	4 [3–5]	18.9%	6.0	11.2	0.69
Average nightly # of SWCs: respondent	960	4 [3–5]	10.2%	4.0	5.6	0.58
# of nightly SWCs use condom with	941	2 [2–3]	2.3%	2.4	3.2	0.49
# of nightly SWCs do not use condom with	940	1 [0–2]		1.4	2.2	0.40
Unable to use condom when wanted to with SWC in past 12 m	962	75.6%	17.0%	74.0%	82.6%	0.65
Respondent asks SWC to share HIV status	961					0.38
Never		32.2%		24.2%	42.4%	
Seldom		14.7%		15.9%	17.6%	
Sometimes		41.6%		46.4%	33.8%	
Often		5.7%		6.5%	3.2%	
Always		5.8%		7.0%	3.0%	
SWCs ask respondent to share HIV status	962					0.95
Never		37.2%		36.9%	34.8%	
Seldom		15.6%		18.6%	18.4%	
Sometimes		40.5%		39.7%	41.5%	
Often		4.4%		3.2%	3.5%	
Always		2.3%		1.6%	1.8%	

*12 m* 12 months, *FSW* female sex worker, *ICC* Intraclass Correlation Coefficient, *IQR* inter-quartile range, *IVR* interviewer, *SWC* sex work client, *ZMW* Zambian Kwacha: 1 Kwacha ~US$ 10

^a^ All bivariate regressions included study site fixed effects and interviewer random intercepts. Values for male and female IVR are marginal predicted values based on regression coefficients. ^b^ ICC is the proportion of all variance in a model without interviewer gender attributable to variation in interviewer identity; not available for Poisson or ordered logistic models.. *P*-value is for a *χ*^2^ test, adjusted for multiple testing across all results shown in Tables 1, 2, 3 and 4 using the Benjamini-Hochberg method

**Table 3:** Sexual health responses by ZEST study population

	Univariate			Bivariate regression^b^		
	N	% / Median [IQR]	ICC ^c^	Male IVR	Female IVR	*p*-value
Age at first sex	960	16 [15–18]	1.6%	16.2	16.2	1.00
Ever been pregnant	965	83.8%	2.3%	86.3%	81.1%	0.40
Number of pregnancies	809	2 [1–3]		2.1	2.2	0.69
Number of living children	803	1 [1–2]		1.6	1.8	0.59
Ever had unwanted pregnancy	809	61.7%	12.2%	60.2%	65.4%	0.84
Of which, trying to avoid pregnancy at the time	497	45.5%	19.5%	43.0%	47.9%	0.89
Ever ended a pregnancy	809	47.7%	20.6%	44.1%	53.2%	0.76
Currently using family planning	964	75.9%	11.6%	79.3%	75.8%	0.87
Has a primary partner	964	59.8%	2.2%	60.4%	59.3%	0.94
Partner knows about sex work	572	16.9%	15.0%	27.2%	23.5%	0.89
Condom use with primary partner	574					0.76
Never		37.3%		40.2%	33.7%	
Seldom		10.1%		11.9%	11.4%	
Sometimes		39.7%		38.7%	43.1%	
Often		2.8%		2.1%	2.6%	
Always		10.1%		7.1%	9.2%	
Likelihood HIV-positive ^a^	956	5 [4–6]	8.0%	4.9	5.4	0.55
Likelihood will contract HIV in the next year ^a^	947	6 [5–8]	6.3%	5.9	5.9	1.00
Likelihood woman acquires HIV from single act ^a^	957	8 [5–10]	22.6%	7.6	6.7	0.51
How likely to take actions to reduce risk of HIV	962					0.76
Very likely		58.7%		57.3%	66.3%	
Somewhat likely		30.9%		33.6%	27.6%	
Unlikely		5.8%		5.2%	3.6%	
Very unlikely		4.6%		3.8%	2.5%	
Estimated proportion of FSW living with HIV ^a^	937	7 [6–9]	5.8%	7.1	7.2	0.86
Estimated proportion of SWC living with HIV ^a^	935	8 [5–9]	4.0%	7.2	7.2	0.99
Knows HIV status of primary partner	574	42.0%	9.3%	40.2%	43.0%	0.92
Ever tested for HIV	958	79.3%	16.3%	84.3%	78.8%	0.75
Months since most recent HIV test	752	6 [4–12]	6.3%	14.1%	9.8%	0.46
Received results of most recent HIV test	745	92.3%	14.4%	94.1%	93.4%	0.95
Needed healthcare but unable to access in past 12 m	963	36.8%	12.7%	35.7%	36.1%	0.99
Comfortable telling medical provider about sex work	963	69.1%	16.8%	73.9%	65.9%	0.69
Feel medical provider judges for sex work	961	36.2%	29.3%	24.1%	47.5%	0.38
Ever been tested for STIs	964	49.1%	0.2%	50.0%	48.5%	0.87
Months since most recent STI test	471	6 [3–12]	7.7%	12.2	9.9	0.69

*FSW* female sex worker, *ICC* Intraclass Correlation Coefficient, *IQR* inter-quartile range, *IVR* interviewer, *SWC* sex work client, *ZMW* Zambian Kwacha: 1 Kwacha ~US$ 10

^a^10-point scale

^b^All bivariate regressions included study site fixed effects and interviewer random intercepts. Values for male and female IVR are marginal predicted values based on regression coefficients. ^c^ ICC is the proportion of all variance in a model without interviewer gender attributable to variation in interviewer identity; not available for Poisson or ordered logistic models.. *P*-value is for a *χ*^2^ test, adjusted for multiple testing across all results shown in Tables 1, 2, 3 and 4 using the Benjamini-Hochberg method

**Table 4:** Other HIV risk factor responses by ZEST study population

	Univariate			Bivariate regression ^a^		
	N	% / Median [IQR]	ICC ^b^	Male IVR	Female IVR	*p*-value
Any physical abuse as child (age < 15)	964	61.0%	29.4%	74.0%	52.4%	0.46
Any physical abuse as adult (age 15+)	965	64.8%	19.9%	76.2%	54.4%	0.26
Of which, from SWC	625	25.0%	27.6%	17.7%	28.8%	0.65
Any physical abuse in past 12 m	964	51.1%	20.0%	49.8%	55.7%	0.87
Of which, from SWC	494	53.6%	36.6%	36.6%	79.4%	**0.02**
Of which, from partner	520	65.6%	35.1%	80.8%	42.4%	0.06
Any sexual abuse as child (age < 15)	963	35.5%	19.7%	36.9%	32.7%	0.89
Any adult sexual abuse (age 15+)	965	44.7%	8.6%	45.6%	43.4%	0.94
Of which, from SWC	431	32.0%	10.8%	28.0%	36.3%	0.67
Any sexual abuse in past 12 m	964	46.6%	23.6%	41.9%	54.1%	0.69
Of which, from SWC	450	60.4%	39.1%	41.9%	83.7%	**0.001**
Of which, from partner	450	42.4%	43.7%	53.2%	22.8%	0.46
Had sex because afraid in past 12 m	965	60.7%	29.8%	55.8%	70.1%	0.58
Of which, with SWC	586	59.0%	37.9%	45.5%	76.5%	**0.04**
Of which, with partner	586	44.7%	37.9%	51.8%	29.0%	0.55
Frequency of alcohol consumption	963					0.76
Never		9.7%		9.3%	6.8%	
Monthly or less		4.6%		4.5%	3.5%	
2–4 times/month		10.8%		11.2%	9.1%	
2–3 times/week		28.6%		31.5%	29.0%	
4+ times/week		46.4%		43.5%	51.7%	
Frequency of having ≥6 drinks in a night	869					0.99
Never		4.3%		3.3%	3.2%	
Monthly or less		5.5%		4.5%	4.5%	
2–4 times/month		7.4%		6.5%	6.4%	
2–3 times/week		40.7%		44.2%	44.0%	
4+ times/week		42.1%		41.4%	41.8%	
Taken any intoxicating substance in past 12 months	963	28.3%	15.7%	24.4%	28.5%	0.87
Taken any non-prescription injected drugs in the past 12 m	963	4.0%	33.1%	1.2%	5.7%	0.19
Ever harassed by police	963	31.4%	4.1%	28.7%	33.9%	0.67
Ever arrested/incarcerated	964	28.5%	11.1%	21.4%	36.3%	0.26
Stood up to someone to help a fellow FSW in past 12 m	965	0.0%	24.3%	83.1%	83.8%	0.99
Attended a public event where identifiable as FSW in past 12 m	964	41.5%	29.9%	35.6%	50.0%	0.67
Feels strong sense of unity with FSWs in general	963	20.4%		18.7%	16.9%	0.94
PHQ-9 raw score (range 0 to 27)	930	9 [6–13]	37.0%	9.4	10.7	0.79
HIV stigma scale raw score (range 0–9)	946	1 [0–1]	9.0%	1.0	0.6	0.38
Adapted FSSQ scale raw score (range 0–30)	956	8 [3–13]	27.5%	8.6	9.3	0.89
Generalized self-efficacy raw score (range 10–40)	932	32.5 [27–38]	34.5%	30.7	32.8	0.67

*12 m* 12 months, *FSSQ* Duke-UNC Functional Social Support Questionnaire, *FSW* female sex worker, *ICC* Intraclass Correlation Coefficient, *IQR* inter-quartile range, *IVR* interviewer, *PHQ-9* Patient Health Questionnaire-9, *SWC* sex work client

^a^ All bivariate regressions included study site fixed effects and interviewer random intercepts. Values for male and female IVR are marginal predicted values based on regression coefficients. ^b^ ICC is the proportion of all variance in a model without interviewer gender attributable to variation in interviewer identity; not available for Poisson or ordered logistic models.. *P*-value is for a *χ*^2^ test, adjusted for multiple testing across all results shown in Tables 1, 2, 3 and 4 using the Benjamini-Hochberg method. *P*-values <0.05 shown in bold

### Statistical analyses

We described how survey responses varied according to the gender of the interviewer, testing for significant differences using Wilcoxon Rank-Sum and χ^2^ tests for continuous/ordinal and nominal categorical data respectively. We then conducted multilevel regression analysis for each outcome, with respondents nested within interviewers, using the appropriate link function for each outcome. We first ran models that contained fixed effects for study site and random intercepts for interviewer identity, and recorded the intraclass correlation coefficient (ICC) at the interviewer level, i.e. the proportion of model variance explained by interviewer identity. ICC was calculable for linear and logistic models, but not for Poisson or ordered logistic ones. Additional file 1: Table S1 details model forms for all variables.

We then ran models with an indicator variable for female gender to test for systematic differences in response by gender of interviewer. We did not adjust for respondent covariates other than study site and interviewer since interviewers were randomly assigned (within study sites), and thus other factors should not change any associations seen between interviewer gender and self-reported variables. From each regression model we estimated prevalences for male and female interviewers based on marginal predicted values from regression coefficients. Given that we were conducting many tests of the same hypothesis, i.e., that responses for each variable differed by interviewer gender, we adjusted all *p*-values for multiple testing using the Benjamini-Hochberg methodology [30]. We conducted a sensitivity analysis modelling all bivariate associations as three-level models additionally including random intercepts for peer educator identity.

Finally, we considered how adjustment for interviewer identity and gender affected measures of association between key covariates (study arm, age, past abuse) and subsequent HIV self-testing, to evaluate whether variation in responses by interviewer gender also affected measures of association between multiple measures. In line with the ZEST primary outcomes analysis [28], we ran generalized linear models with a Poisson distribution and log link, and standard errors robust to heteroskedasticity. For each combination of exposure (study arm, age in categories – < 25, 25–29, 30–34, ≥35 – and baseline reports of adult physical and sexual abuse) and outcome (recent HIV testing at one and 4 months), we ran three models: (i) just containing fixed effects for study arm and site; (ii) adding random effects for interviewer identity; and (iii) adding a fixed effect for female vs. male interviewer. Statistical analyses were run in Stata version 13 (College Station, TX).

## Results

The ZEST baseline sample consisted of 965 FSWs interviewed by 9 male and 7 female interviewers (all with 60 respondents bar one with 65). There were equal numbers of male and female interviewers in Chirundu (two of each), more female than male in Kapiri Mposhi (three vs one) and more male than female in Livingstone (six vs two). Interviewer ages ranged from 25 to 45 (median: 35, interquartile range [IQR]: 31.5–38). The socio-demographic and behavioural composition of the population has been described previously [28], but briefly they were young (75% aged under 30), almost none were married and they had low SES (Table 1).

For the 62 variables using linear and logistic regression, models containing only fixed effects for study site and random intercepts for interviewer identity, variability at the interviewer level accounted a median of 14.6% of all variance (IQR: 7.6–23.4%). Interviewer-level variation was generally lowest for socio-demographic and cognitively simple questions, and highest for questions relating to sexual behaviour, substance use, abuse and psychosocial wellbeing (Tables 1, 2, 3 and 4).

### Socio-demographics

FSWs were more likely to report lower educational attainment and lower income to female interviewers than to male ones (Table 1). Specifically, respondents were less likely to tell female interviewers that they were literate, more likely to report earning less than ZMW 500 (~US$50) per month and more likely to report being poor or very poor; this last comparison was statistically significant after adjusting for multiple testing. Despite these differences, FSWs reported almost identical levels of self-perceived relative SES to male and female interviewers.

### Sex work

In the context of sex work, FSWs were non-significantly more likely to report they always asked clients to use condoms, and less likely to report that they frequently asked clients to disclose their HIV status, to male interviewers (Table 2). Respondents told female interviewers that other FSWs had more clients per night than they did male interviewers, although much of this difference was due to a few outlying values for one interviewer.

### Sexual behaviour and health

When discussing their sexual health and behaviour other than sex work, respondents reported very similar behaviours and beliefs to male and female interviewers (Table 3).

One exception to this was that FSWs were non-significantly more likely to tell female interviewers that they were uncomfortable telling medical providers about sex work and that they felt judged by medical providers for doing sex work.

### Other HIV risk factors

**Fig. 1:**
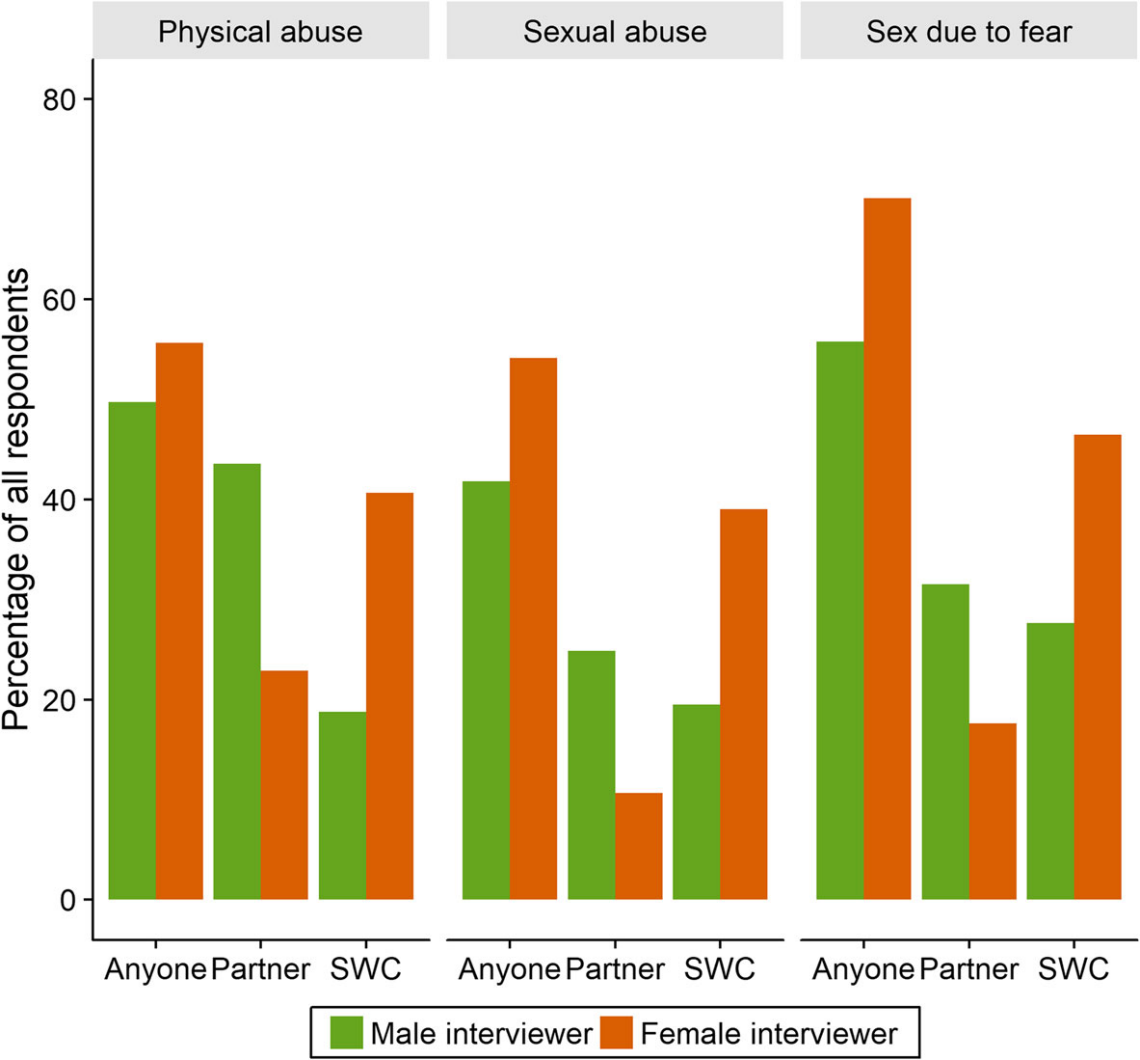
Proportion of ZEST respondents reporting experiencing violence in the past 12 months from anyone and from sex work clients specifically. SWC: sex work client; Partner: any non-client sexual partner

Reporting patterns for substance use, FSW-empowerment and various psychosocial scales were very similar by interviewer (Table 4). However, reporting of abuse varied substantially by interviewer gender. Specifically, FSWs reported non-significantly, but substantially, lower rates of lifetime childhood or adult physical abuse to female interviewers (over 20 percentage points difference), but similar rates of sexual abuse at both ages. When asked specifically about abuse in the past 12 months, respondents reported significantly higher rates of both physical and sexual abuse from sex work clients to female respondents, and correspondingly lower rates of abuse from their non-client partners. They were similarly far (over 30 percentage points) more likely to report having had sex with a client in the past 12 months because they were afraid to female interviewers (Fig. 1). Additional adjustment for peer educator identity did substantively affect any of the above results (Additional file 1: Table S3).

### Associations

In regressions predicting recent HIV testing history at follow-up, we did not see any effect of adding interviewer random effects or respondent age to models of the primary ZEST study association, i.e. difference in testing rates by study arm (Table 5). Nor did we see any impact of accounting for interviewer identity on the association between history of sexual abuse and recent HIV testing at 1 month. However at 4 months, sexual abuse was significantly associated with not testing when no adjustment was made for interviewer identity, but this became non-significant after including interviewer random effects. Including interviewer gender did not affect our associations of interest, over and above interviewer random effects.

**Table 5:** Impact of interviewer identity on associations between baseline covariates and HIV testing history in ZEST trial

	Month 1 follow-up, tested in past 30 days						Month 4 follow-up, tested in past 30 days					
1. No covariates												
Study arm A	1.00		1.00		1.00		1.00		1.00		1.00	
Study arm B	1.07	[1.02–1.12]	1.07	[0.94–1.21]	1.07	[0.95–1.20]	1.11	[1.03–1.21]	1.13	[0.98–1.31]	1.14	[0.98–1.31]
Study arm C	0.95	[0.89–1.01]	0.95	[0.84–1.07]	0.94	[0.85–1.05]	1.06	[0.97–1.15]	1.07	[0.95–1.21]	1.08	[0.96–1.22]
Female vs. male interviewer					0.97	[0.91–1.04]					1.39	[0.70–2.73]
Interviewer random effects			Yes		Yes				Yes		Yes	
2. Age												
18–24	1.00		1.00		1.00		1.00		1.00		1.00	
25–29	0.96	[0.92–1.02]	0.96	[0.91–1.03]	0.96	[0.91–1.03]	1.00	[0.93–1.08]	1.01	[0.93–1.10]	1.01	[0.93–1.10]
30–34	0.94	[0.88–1.01]	0.94	[0.87–1.02]	0.94	[0.87–1.02]	0.99	[0.91–1.08]	0.97	[0.91–1.04]	0.97	[0.91–1.04]
35+	0.94	[0.87–1.01]	0.94	[0.85–1.03]	0.94	[0.85–1.03]	0.92	[0.81–1.05]	0.91	[0.80–1.03]	0.91	[0.80–1.03]
Female vs. male interviewer					0.97	[0.91–1.04]					1.39	[0.70–2.74]
Interviewer random effects			Yes		Yes				Yes		Yes	
3. Sexual abuse												
Any sexual abuse as adult	1.01	[0.97–1.06]	1.01	[0.97–1.06]	1.01	[0.97–1.06]	0.92	[0.86–0.98]	0.98	[0.90–1.05]	0.97	[0.90–1.05]
Female vs. male interviewer					0.97	[0.91–1.04]					1.38	[0.70–2.72]
Interviewer random effects			Yes		Yes				Yes		Yes	

This table summarizes results from 18 regressions (nine for each outcome). Full regression output is provided in Additional file 1: Table S2

All regressions are generalized linear models with a Poisson distribution and log link, and robust standard errors. All contain fixed effects for study arm (even when not shown here) and site. Month 1 *N* = 884; month 4 *N* = 892

## Discussion

In this analysis of data from an HIV self-test trial among FSWs in three Zambian border towns, we show that interviewers often substantially affected what respondents reported regarding their lives, in particular their psychological wellbeing and experiences of violence. In the context of 16 interviewers each conducting at least 60 interviews, an average of one-sixth of all variance in question responses was observed at the interviewer level, even after accounting for study site. This interviewer-level variance rose to almost one-third for questions about psychological ill-health and violence, despite the prevalence of both being very high and careful interviewer training [27]. These variations fed through in some cases to measures of association, i.e., failing to account for interviewer effects led to different coefficient estimates in regression models.

The importance of interviewer variation has long been recognized in the survey design and analysis literature [12, 19, 31] and our findings reinforce the importance of interviewers for measures of prevalence. Our findings support particularly strong interviewer effects for sensitive topics, notably physical and sexual abuse, and subjective ones, such as depression, social support and self-efficacy. For example, for the question “In the past 12 months, has a sexual partner ever physically forced you to have sex when you did not want to?”, the proportion of each interviewer’s 60 respondents answering in the affirmative varied from 13 to 97%. This occurred despite the two interviewers with the most extreme proportions working in the same town, and thus theoretically interviewing fully exchangeable respondents.

The potential impact of interviewer variation can be minimized by careful training in question presentation, and monitoring of response patterns by interviewer identity during study conduct (with feedback of these findings to the field teams). Other potentially useful steps include matching interviewers and respondents by age and gender, and providing support for interviewers in managing their own distress in hearing reports of violence or other hardship [23]. When interviewer-level variance is anticipated, it is also preferable to have a large number of interviewers doing few interviews, rather than a few interviewers doing many; this both reduces the burden on interviewers, and avoids outlying interviewers from having oversized impacts [32].

Despite the substantial variance in responses at the interviewer level, interviewers’ gender was associated with relatively few variables. There were substantive (i.e., more than 10 percentage points), if non-significant, differences by gender-of-interviewer for several variables and significant differences for two question topics: SES and sex-work related violence. We were unable to determine in this analysis whether the gender-of-interviewer differences seen reflect social distance or social desirability, since there was no variation in respondent gender. However, our finding that the largest gender-of-interviewer effects exist for topics which have substantial gender components (i.e., SES and IPV) provides support for social role theory. Specifically, FSWs reported having lower SES and more recent sex-work related IPV to female interviewers. This was in contrast to almost no reporting difference for questions such as age, marital status, pregnancy history and perceived risk of being HIV-positive. These findings highlight that, while matching interviewers and respondents on key characteristics may not be feasible, the influence of interviewer-respondent dyad characteristics should evaluated for analysis on topics with strong social role expectations, such as gender-based violence and economic behaviour.

We also showed that the association between two self-reported variables can be confounded by interviewers. In our analysis, recent HIV testing behaviour was significantly negatively associated with both past physical and sexual abuse when we did not include interviewer identity in our models, but this association was attenuated and rendered non-significnat by including interviewer-level random effects. In order for interviewers to have such an effect, both exposure and outcome must be susceptible to interviewer influence. This is clearly the case when both variables are self-reported, but can also arise when interviewers are also asking individuals to take a test – a topic that has been substantively investigated in the context of HIV testing within population studies [33, 34]. Our results highlight the need to consider interviewer identity as a possible confounder in associational as well as prevalence analyses.

Given that much of the data in this study is self-reported, it is difficult to know which interviewers are receiving the “truer” responses and thus which results to act on. In this population, for example, even based on responses to male interviewers respondents are poor and at substantial risk for IPV: median income is under $600 per annum, half the Zambian average, and over 40% reported each of: physical abuse; sexual abuse; and having had sex when they did not want to because they were afraid in the past 12 months. There is clearly a substantial public health concern whichever values are closer to reality. However, in some other settings, the level of impact interviewer gender had in this study may be sufficient to provide conflicting results – with male interviewers finding a substantial health risk but female interviewers only a limited one, or vice versa.

### Strengths and limitations

Our results should be interpreted in the light of various strengths and limitations. The underlying ZEST study comprised almost 1000 FSWs who were part of a population with relatively little experience of engaging with researchers, which should minimize respondent learning effects in terms of intentional mis-reporting. However, this may also have led to respondents misunderstanding questions they had not previously considered in a systematic fashion.

Since all ZEST participants were women, we are unable to differentiate whether the gender effects we saw reflected gender-of-interviewer effects or gender-homophily of interviewer-respondent dyads. Our ability to generalize from the ZEST study population to others is also somewhat limited: it is hard to know whether FSWs in more cosmopolitan settings, or women more generally in Zambia or sub-Saharan Africa (including those engaging in informal sex work), would have been similarly affected by interviewer characteristics. Nevertheless, our key findings that interviewers can generate substantial, systematic differences in item response patterns, even when randomly assigned to respondents, are likely to be widely applicable.

Furthermore, we do not have sufficiently detailed information available on interviewer identities to determine whether interviewers varied systematically by gender on other characteristics, e.g. educational attainment, that might have affected their ability to elicit sensitive responses from respondents. Concern on this front is somewhat allayed by the very similar responses (and low ICC values) for less sensitive topics. Finally, the ZEST study did not include follow-up interviews on the topic of interviewer-respondent interaction, and thus we are not able to directly assess whether between-interviewer differences reflected true random difference or some combination of social distance, social desirability and social role.

## Conclusions

In a trial of HIV self-testing among FSWs in Zambian border towns, we found very high levels of interviewer-level variability in responses to sensitive questions. We also found some evidence of differential reports by interviewer gender for topics relating to gender roles, and demonstrated that interviewers influenced measures of association between a key risk factor, past sexual abuse, and the study’s primary outcome, recent HIV testing at follow-up visits. This work highlights the importance of conducting careful interviewer training, and evaluating how responses vary by interviewer, for sensitive questions – especially when prevalence or association measures have policy relevance. It also underscores the importance of considering social distance between respondents and interviewers, especially for topics that are either highly stigmatized or have strong social role expectations.

## Additional file

Additional file 1: **Table S1**. Regression forms for variables considered. **Table S2**. Impact of interviewer identity on associations between baseline covariates and HIV testing history in an HIV self-test trial amongst female sex workers in Zambia, full regression results. **Table S3**. Comparison of bivariate associations from hierarchical models containing random intercepts only for interviewers (two-level) or interviewers and peer educators (three-level). (PDF 120 kb)

## Abbreviations

AIDS: Acquired Immunodeficiency Syndrome
CAPI: Computer-assisted personal interview
FSW: Female sex worker
GBV: Gender-based violence
HIV: Human Immunodeficiency virus
ICC: Intraclass correlation coefficient
IPV: Intimate partner violence
IQR: Interquartile range
SES: Socioeconomic status
USA: United States of America
ZEST: Zambian Peer Educators for HIV Self-Testing study
ZMW: Zambian Kwacha
