# Supplementary material for: The influence of interviewers on survey responses among female sex workers in Zambia
Source: BMC Med Res Methodol. 2019 Mar 15;19:60. doi: 10.1186/s12874-019-0703-2 (PMC6419821; doi:10.1186/s12874-019-0703-2)
Supplement: Supplementary file 1 — Table S1. Regression forms for variables considered. Table S2. Impact of interviewer identity on associations between baseline covariates and HIV testing history in an HIV self-test trial amongst female sex workers in Zambia, full regression results. Table S3. Comparison of bivariate associations from hierarchical models containing random intercepts only for interviewers (two-level) or interviewers and peer educators (three-level). (PDF 120 kb) [file 12874_2019_703_MOESM1_ESM.pdf]

SUPPLEMENTARY MATERIALS

Title: The influence of interviewers on survey responses among female sex workers in Zambia

Supplementary Table 1: Regression forms for variables considered

Variable	Regression form used
Age	Linear
Ever married	Logistic
Currently divorced/separated	Logistic
Education	Ordered logistic
Able to read and write	Logistic
Monthly income (ZMW)	Ordered logistic
Financial situation	Ordered logistic
Mobile phone ownership	Logistic
Self-perceived relative SES	Poisson
Any income from non-sex-work	Logistic
Age at first sex for money	Linear
Condoms available while working	Ordered logistic
Ask SWC to use a condom	Ordered logistic
SWCs ask to use a condom	Ordered logistic
SWCs request that not to use a condom	Ordered logistic
ZMW price for vaginal sex with condom	Linear
ZMW price for vaginal sex without condom	Linear
ZMW price for anal sex with condom	Linear
ZMW price for anal sex without condom	Linear
Average nightly # of SWCs: other FSW	Linear
Average nightly # of SWCs: respondent	Linear
# of nightly SWCs use condom with	Linear
# of nightly SWCs do not use condom with	Poisson
Unable to use condom when wanted to with SWC in past 12 months	Logistic
Respondent asks SWC to share HIV status	Ordered logistic
SWCs ask respondent to share HIV status	Ordered logistic
Age at first sex	Linear
Ever been pregnant	Logistic
Number of pregnancies	Poisson
Number of living children	Poisson
Ever had unwanted pregnancy	Logistic
Of which, trying to avoid pregnancy at the time	Logistic
Ever ended a pregnancy	Logistic
Currently using family planning	Logistic
Has a primary partner	Logistic
Partner knows about sex work	Logistic
Condom use with primary partner	Ordered logistic
Likelihood HIV-positive	Linear
Likelihood will contract HIV in the next year	Linear
Likelihood woman acquires HIV from single act	Linear
How likely to take actions to reduce risk of HIV	Ordered logistic
Estimated proportion of FSW living with HIV	Linear
Estimated proportion of SWC living with HIV	Linear
Knows HIV status of primary partner	Logistic
Ever tested for HIV	Logistic
Months since most recent HIV test	Linear
Received results of most recent HIV test	Logistic
Needed healthcare but unable to access in past 12 months	Logistic
Comfortable telling medical provider about sex work	Logistic
Feel medical provider judges for sex work	Logistic
Ever been tested for STIs	Logistic
Months since most recent STI test	Linear

Variable	Regression form used
Any physical abuse as child (age <15)	Logistic
Any physical abuse as adult (age 15+)	Logistic
Of which, from SWC	Logistic
Any physical abuse in past 12 months	Logistic
Of which, from SWC	Logistic
Of which, from partner	Logistic
Any sexual abuse as child (age <15)	Logistic
Any adult sexual abuse (age 15+)	Logistic
Of which, from SWC	Logistic
Any sexual abuse in past 12 months	Logistic
Of which, from SWC	Logistic
Of which, from partner	Logistic
Had sex because afraid in past 12 months	Logistic
Of which, with SWC	Logistic
Of which, with partner	Logistic
Frequency of alcohol consumption	Ordered logistic
Frequency of having ≥6 drinks in a night	Ordered logistic
Taken any intoxicating substance in past 12 months	Logistic
Taken any non-prescription injected drugs in the past 12 months	Logistic
Ever harassed by police	Logistic
Ever arrested/incarcerated	Logistic
Stood up to someone to help a fellow FSW in past 12 months	Logistic
Attended a public event where identifiable as FSW in past 12 months	Logistic
Feels strong sense of unity with FSWs in general	Ordered logistic
PHQ-9 raw score (range 0 to 27)	Linear
HIV stigma scale raw score (range 0-9)	Linear
Adapted FSSQ scale raw score (range 0-30)	Linear
Generalized self-efficacy raw score (range 10-40)	Linear

Supplementary Table 2: Impact of interviewer identity on associations between baseline covariates and HIV testing history in an HIV self-test trial amongst female sex workers in Zambia, full regression results

	Month 1 follow-up, tested in past 30 days						Month 4 follow-up, tested in past 30 days					
**1. No covariates**												
Study arm A	1.00		1.00		1.00		1.00		1.00		1.00	
Study arm B	1.07	[0.99 - 1.15]	1.07	[0.90 - 1.26]	1.07	[0.90 - 1.26]	1.11	[0.98 - 1.26]	1.13	[0.94 - 1.36]	1.14	[0.94 - 1.37]
Study arm C	0.95	[0.86 - 1.05]	0.95	[0.80 - 1.13]	0.94	[0.79 - 1.12]	1.06	[0.92 - 1.21]	1.07	[0.89 - 1.30]	1.08	[0.90 - 1.31]
Livingstone	1.00		1.00		1.00		1.00		1.00		1.00	
Kapiri	0.83	[0.72 - 0.94]	0.83	[0.69 - 0.99]	0.84	[0.69 - 1.02]	0.86	[0.78 - 0.96]	0.85	[0.52 - 1.39]	0.72	[0.44 - 1.20]
Chirundu	1.10	[1.04 - 1.15]	1.10	[0.93 - 1.29]	1.10	[0.93 - 1.31]	0.79	[0.68 - 0.93]	0.67	[0.40 - 1.11]	0.62	[0.38 - 1.02]
Female vs. male interviewer					0.97	[0.83 - 1.14]					1.39	[0.91 - 2.11]
Interviewer random effects	1.00		1.00		1.00		1.00		1.00		1.00	
**2. Age**												
Study arm A	1.00		1.00		1.00		1.00		1.00		1.00	
Study arm B	1.07	[1.02 - 1.12]	1.07	[0.95 - 1.20]	1.07	[0.95 - 1.20]	1.11	[1.03 - 1.21]	1.13	[0.98 - 1.30]	1.14	[0.98 - 1.31]
Study arm C	0.95	[0.89 - 1.01]	0.95	[0.84 - 1.06]	0.94	[0.85 - 1.05]	1.05	[0.97 - 1.15]	1.07	[0.95 - 1.20]	1.08	[0.97 - 1.21]
Livingstone	1.00		1.00		1.00		1.00		1.00		1.00	
Kapiri	0.82	[0.76 - 0.89]	0.82	[0.64 - 1.07]	0.84	[0.66 - 1.05]	0.86	[0.80 - 0.94]	0.85	[0.71 - 1.03]	0.73	[0.48 - 1.11]
Chirundu	1.10	[1.06 - 1.13]	1.10	[1.03 - 1.17]	1.11	[1.04 - 1.17]	0.80	[0.73 - 0.88]	0.67	[0.21 - 2.16]	0.62	[0.18 - 2.16]
Age18 - 24	1.00		1.00		1.00		1.00		1.00		1.00	
Age25 - 29	0.96	[0.92 - 1.02]	0.96	[0.91 - 1.03]	0.96	[0.91 - 1.03]	1.00	[0.93 - 1.08]	1.01	[0.93 - 1.10]	1.01	[0.93 - 1.10]
Age30 - 34	0.94	[0.88 - 1.01]	0.94	[0.87 - 1.02]	0.94	[0.87 - 1.02]	0.99	[0.91 - 1.08]	0.97	[0.91 - 1.04]	0.97	[0.91 - 1.04]
Age35+	0.94	[0.87 - 1.01]	0.94	[0.85 - 1.03]	0.94	[0.85 - 1.03]	0.92	[0.81 - 1.05]	0.91	[0.80 - 1.03]	0.91	[0.80 - 1.03]
Female vs. male interviewer					0.97	[0.91 - 1.04]					1.39	[0.70 - 2.74]
Interviewer random effects	1.00		1.00		1.00		1.00		1.00		1.00	

	Month 1 follow-up, tested in past 30 days						Month 4 follow-up, tested in past 30 days					
**3. Physical abuse**												
Study arm A	1.00		1.00		1.00		1.00		1.00		1.00	
Study arm B	1.07	[1.02 - 1.12]	1.07	[0.94 - 1.21]	1.07	[0.95 - 1.20]	1.12	[1.04 - 1.21]	1.13	[0.98 - 1.31]	1.14	[0.98 - 1.32]
Study arm C	0.95	[0.89 - 1.01]	0.95	[0.84 - 1.07]	0.94	[0.85 - 1.05]	1.07	[0.98 - 1.16]	1.08	[0.95 - 1.21]	1.08	[0.96 - 1.22]
Livingstone	1.00		1.00		1.00		1.00		1.00		1.00	
Kapiri	0.83	[0.76 - 0.89]	0.83	[0.63 - 1.08]	0.84	[0.66 - 1.06]	0.85	[0.78 - 0.92]	0.85	[0.71 - 1.02]	0.72	[0.48 - 1.10]
Chirundu	1.09	[1.06 - 1.13]	1.09	[1.03 - 1.17]	1.10	[1.04 - 1.17]	0.79	[0.72 - 0.87]	0.67	[0.21 - 2.14]	0.62	[0.18 - 2.14]
Any physical abuse as adult	1.00	[0.95 - 1.05]	1.00	[0.93 - 1.07]	0.99	[0.93 - 1.07]	0.90	[0.84 - 0.96]	0.97	[0.93 - 1.02]	0.98	[0.94 - 1.02]
Female vs. male interviewer					0.97	[0.90 - 1.05]					1.38	[0.70 - 2.73]
Interviewer random effects			Yes		Yes				Yes		Yes	
**4. Sexual abuse**												
Study arm A	1.00		1.00		1.00		1.00		1.00		1.00	
Study arm B	1.07	[1.02 - 1.12]	1.07	[0.94 - 1.21]	1.07	[0.95 - 1.20]	1.12	[1.03 - 1.21]	1.13	[0.98 - 1.31]	1.14	[0.98 - 1.31]
Study arm C	0.95	[0.89 - 1.01]	0.95	[0.84 - 1.07]	0.94	[0.85 - 1.05]	1.05	[0.97 - 1.15]	1.07	[0.95 - 1.21]	1.08	[0.96 - 1.22]
Livingstone	1.00		1.00		1.00		1.00		1.00		1.00	
Kapiri	0.83	[0.76 - 0.89]	0.83	[0.63 - 1.08]	0.84	[0.66 - 1.06]	0.86	[0.79 - 0.94]	0.85	[0.71 - 1.02]	0.73	[0.48 - 1.10]
Chirundu	1.09	[1.06 - 1.13]	1.09	[1.03 - 1.16]	1.10	[1.04 - 1.17]	0.80	[0.73 - 0.88]	0.67	[0.21 - 2.16]	0.62	[0.18 - 2.15]
Any sexual abuse as adult	1.01	[0.97 - 1.06]	1.01	[0.97 - 1.06]	1.01	[0.97 - 1.06]	0.92	[0.86 - 0.98]	0.98	[0.90 - 1.05]	0.97	[0.90 - 1.05]
Female vs. male interviewer					0.97	[0.91 - 1.04]					1.38	[0.70 - 2.72]
Interviewer random effects			Yes		Yes				Yes		Yes	

This table provides full results from 24 regressions (12 for each outcome).
All regressions are generalized linear models with a Poisson distribution and log link, and robust standard errors. Month 1 N=884; month 4 N=892.

Supplementary Table 3: Comparison of bivariate associations from hierarchical models containing random intercepts only for interviewers (two-level) or interviewers and peer educators (three-level)

	Two-level models				Three-level models			
	ICC [b]	Male IVR	Female IVR	p-value	ICC [b]	Male IVR	Female IVR	p-value
Age	3.9%	26.2	26.3	0.99	2.5%	26.2	26.2	1.00
Ever married	12.5%	68.7%	75.5%	0.69	12.2%	69.4%	76.4%	0.69
Currently divorced/separated	10.4%	26.8%	18.4%	0.55	10.0%	25.9%	17.3%	0.54
Education				0.65				0.64
No formal education		7.3%	11.8%			5.5%	9.7%	
Primary (up to 9 years)		44.6%	53.0%			46.8%	56.8%	
> 9 years		48.1%	35.2%			47.7%	33.5%	
Able to read and write	9.1%	82.8%	67.7%	0.060	8.2%	84.7%	69.2%	0.055
Income (ZMW)				0.38				0.34
No income		2.6%	24.8%			1.5%	21.5%	
<250 kwacha		9.9%	38.4%			8.4%	44.5%	
251-500 kwacha		39.1%	29.6%			40.6%	28.8%	
501-1000 kwacha		37.4%	6.2%			40.5%	4.7%	
1001-1500 kwacha		6.9%	0.6%			6.1%	0.4%	
>1500 kwacha		4.2%	0.4%			2.9%	0.2%	
Financial situation				0.020				0.020
Very poor		4.8%	24.1%			4.1%	23.0%	
Poor		32.8%	54.1%			32.7%	56.0%	
Just getting by		46.5%	18.9%			48.3%	18.6%	
Comfortable		14.2%	2.6%			13.3%	2.2%	
Very comfortable		1.8%	0.3%			1.6%	0.2%	
Mobile phone ownership	6.3%	86.4%	85.2%	0.94	4.5%	88.7%	87.6%	0.93
Self-perceived relative SES (10 point scale)		3.1	3.0	0.94		3.1	2.9	0.94
Any income from non-sex-work	7.5%	30.6%	27.3%	0.87	7.1%	29.7%	26.3%	0.87
Age at first sex for money	1.6%	19.8	19.0	0.38	0.9%	19.8	19.0	0.34
Condoms available while working				0.26				0.29
Never		1.2%	2.1%			0.9%	1.6%	
Seldom		3.2%	5.3%			2.5%	4.4%	
Sometimes		60.7%	68.3%			63.4%	71.7%	
Often		9.7%	7.7%			10.4%	7.9%	
Always		25.1%	16.6%			22.7%	14.4%	
Ask SWC to use a condom				0.55				0.54
Never		2.2%	4.0%			1.5%	2.9%	
Seldom		3.7%	6.4%			2.9%	5.4%	
Sometimes		53.2%	62.2%			56.1%	66.5%	
Often		14.5%	11.0%			15.7%	11.3%	
Always		26.5%	16.4%			23.8%	13.8%	
SWCs ask to use a condom				0.49				0.49
Never		16.2%	7.8%			15.9%	7.6%	
Seldom		24.7%	15.8%			24.8%	15.7%	
Sometimes		54.7%	66.7%			55.1%	67.3%	
Often		2.6%	5.6%			2.5%	5.4%	
Always		1.8%	4.1%			1.7%	4.0%	
SWCs request that not to use a condom				0.46				0.45
Never		10.3%	4.6%			9.8%	4.2%	
Seldom		19.1%	10.9%			19.0%	10.6%	
Sometimes		55.4%	54.5%			56.8%	55.9%	
Often		12.7%	24.1%			12.2%	23.9%	
Always		2.4%	5.8%			2.2%	5.4%	
ZMW price for vaginal sex with condom	6.6%	101	99	0.95	5.4%	102	99	0.94
ZMW price for vaginal sex without condom	7.0%	203	177	0.58	5.8%	205	178	0.54
ZMW price for anal sex with condom	16.7%	146	185	0.65	15.5%	145	184	0.64
ZMW price for anal sex without condom	14.8%	242	263	0.89	12.7%	242	259	0.92
Average nightly # of SWCs: other FSW	18.9%	6.0	11.2	0.69	18.7%	6.1	11.4	0.69
Average nightly # of SWCs: respondent	10.2%	4.0	5.6	0.58	10.1%	4.0	5.6	0.58
# of nightly SWCs use condom with	2.3%	2.4	3.2	0.49	2.2%	2.4	3.2	0.49
# of nightly SWCs do not use condom with		1.4	2.2	0.40		1.3	1.8	0.26

	Two-level models				Three-level models			
	ICC [b]	Male IVR	Female IVR	p-value	ICC [b]	Male IVR	Female IVR	p-value
Unable to use condom when wanted with client in past 12m	17.0%	74.0%	82.6%	0.65	16.4%	75.1%	83.7%	0.64
Respondent asks SWC to share HIV status				0.38				0.37
Never		24.2%	42.4%			23.6%	42.2%	
Seldom		15.9%	17.6%			16.3%	18.1%	
Sometimes		46.4%	33.8%			47.1%	33.9%	
Often		6.5%	3.2%			6.4%	3.1%	
Always		7.0%	3.0%			6.6%	2.8%	
SWCs asks respondent to share HIV status				0.95				0.97
Never		36.9%	34.8%			36.2%	34.5%	
Seldom		18.6%	18.4%			19.2%	19.1%	
Sometimes		39.7%	41.5%			40.1%	41.6%	
Often		3.2%	3.5%			3.0%	3.2%	
Always		1.6%	1.8%			1.5%	1.6%	
Age at first sex	1.6%	16.2	16.2	1.00	1.2%	16.2	16.2	1.00
Ever been pregnant	2.3%	86.3%	81.1%	0.40	1.4%	87.5%	82.4%	0.39
Number of pregancies		2.1	2.2	0.69		2.1	2.2	0.69
Number of living children		1.6	1.8	0.59		1.6	1.8	0.59
Ever had unwanted pregnancy	12.2%	60.2%	65.4%	0.84	12.0%	60.8%	66.5%	0.83
Of which, trying to avoid pregnancy at the time	19.5%	43.0%	47.9%	0.89	19.5%	43.0%	47.9%	0.90
Ever ended a pregnancy	20.6%	44.1%	53.2%	0.76	20.7%	43.5%	53.3%	0.76
Currently using family planning	11.6%	79.3%	75.8%	0.87	11.1%	80.9%	77.3%	0.87
Has a primary partner	2.2%	60.4%	59.3%	0.94	1.1%	61.6%	60.4%	0.94
Partner knows about sex work	15.0%	27.2%	23.5%	0.89	14.6%	25.6%	22.0%	0.90
Condom use with primary partner				0.76				0.76
Never		40.2%	33.7%			40.2%	33.7%	
Seldom		11.9%	11.4%			11.9%	11.4%	
Sometimes		38.7%	43.1%			38.7%	43.1%	
Often		2.1%	2.6%			2.1%	2.6%	
Always		7.1%	9.2%			7.1%	9.2%	
Likelihood HIV-positive (10 point scale)	8.0%	4.9	5.4	0.55	7.6%	4.9	5.4	0.54
Likelihood will contract HIV in the next year (10 point scale)	6.3%	5.9	5.9	1.00	5.5%	5.9	5.9	1.00
Likelihood woman acquires HIV from single act (10 point scale)	22.6%	7.6	6.7	0.51	21.8%	7.6	6.7	0.52
How likely to take actions to reduce risk of HIV				0.76				0.76
Very likely		57.3%	66.3%			58.4%	67.9%	
Somewhat likely		33.6%	27.6%			34.5%	27.6%	
Unlikely		5.2%	3.6%			4.3%	2.8%	
Very unlikely		3.8%	2.5%			2.8%	1.7%	
Estimated proportion of FSW living with HIV (10 point scale)	5.8%	7.1	7.2	0.86	5.8%	7.1	7.2	0.86
Estimated proportion of SWC living with HIV (10 point scale)	4.0%	7.2	7.2	0.99	3.8%	7.2	7.2	0.99
Knows HIV status of primary partner	9.3%	40.2%	43.0%	0.92	8.6%	39.6%	42.3%	0.93
Ever tested for HIV	16.3%	84.3%	78.8%	0.75	13.2%	89.3%	84.3%	0.74
Months since last HIV test	6.3%	14.1	9.8	0.46	6.0%	14.5	9.9	0.45
Received results of most recent HIV test	14.4%	94.1%	93.4%	0.95	11.7%	95.3%	94.8%	0.94
Needed healthcare but unable to access in past 12 months	12.7%	35.7%	36.1%	0.99	12.1%	34.4%	34.7%	1.00
Comfortable telling medical provider about sex work	16.8%	73.9%	65.9%	0.69	16.5%	74.7%	66.3%	0.69
Feel medical provider judges for sex work	29.3%	24.1%	47.5%	0.38	29.2%	23.0%	47.3%	0.37
Ever been tested for STIs	0.2%	49.9%	48.0%	0.87	0.0%	49.9%	47.9%	0.87
Months since last tested for STIs	7.7%	12.2	9.9	0.69	7.3%	12.2	9.9	0.69
Any physical abuse as child (age <15)	29.4%	74.0%	52.4%	0.46	29.0%	75.5%	52.6%	0.46
Any physical abuse as adult (age 15+)	19.9%	76.2%	54.4%	0.26	19.7%	76.8%	54.5%	0.26
Of which, from SWC	27.6%	17.7%	28.8%	0.65	26.5%	15.9%	27.9%	0.64
Any physical abuse in past 12m	20.0%	49.8%	55.7%	0.87	19.8%	49.7%	55.9%	0.87
Of which, from SWC	36.6%	36.6%	79.4%	0.020	36.5%	35.7%	80.4%	0.020
Of which, from partner	35.1%	80.8%	42.4%	0.058	35.1%	83.5%	41.2%	0.051
Any sexual abuse as child (age <15)	19.7%	36.9%	32.7%	0.89	19.6%	36.4%	32.0%	0.90
Any adult sexual abuse (age 15+)	8.6%	45.6%	43.4%	0.94	8.4%	45.4%	43.1%	0.94
Of which, from SWC	10.8%	28.0%	36.3%	0.67	10.8%	28.0%	36.3%	0.68
Any sexual abuse in past 12m	23.6%	41.9%	54.1%	0.69	23.4%	41.3%	54.3%	0.68
Of which, from SWC	39.1%	41.9%	83.7%	0.001	38.7%	41.7%	84.3%	0.002
Of which, from partner	43.7%	53.2%	22.8%	0.46	43.1%	53.2%	21.5%	0.45
Had sex because afraid in past 12m	21.8%	55.8%	70.1%	0.58	21.6%	56.1%	70.9%	0.58
Of which, with SWC	29.8%	45.5%	76.5%	0.040	29.5%	45.5%	76.9%	0.040

	Two-level models				Three-level models			
	ICC [b]	Male IVR	Female IVR	p-value	ICC [b]	Male IVR	Female IVR	p-value
Of which, with partner	37.9%	51.8%	29.0%	0.55	37.7%	51.8%	27.5%	0.54
Frequency of alcohol consumption				0.76				0.73
Never		9.3%	6.8%			8.0%	5.6%	
Monthly or less		4.5%	3.5%			4.3%	3.1%	
2-4 times/month		11.2%	9.1%			11.2%	8.7%	
2-3 times/week		31.5%	29.0%			33.6%	30.5%	
4+ times/week		43.5%	51.7%			43.0%	52.1%	
Frequency of having ≥6 drinks in a night				0.99				1.00
Never		3.3%	3.2%			2.7%	2.7%	
Monthly or less		4.5%	4.5%			4.0%	4.0%	
2-4 times/month		6.5%	6.4%			6.1%	6.1%	
2-3 times/week		44.2%	44.0%			46.5%	46.4%	
4+ times/week		41.4%	41.8%			40.8%	40.8%	
Taken any intoxicating substance in past 12 months	15.7%	24.4%	28.5%	0.87	14.8%	21.7%	26.1%	0.87
Taken any non-prescription injected drugs in the past 12m	33.1%	1.2%	5.7%	0.19	27.6%	0.7%	3.9%	0.20
Ever harassed by police	4.1%	28.7%	33.9%	0.67	3.4%	27.4%	32.8%	0.68
Ever arrested/incarcerated	11.1%	21.4%	36.3%	0.26	10.4%	19.8%	35.2%	0.26
Stood up to someone to help a fellow FSW in past 12m	24.3%	83.1%	83.8%	0.99	23.5%	84.3%	85.0%	0.99
Attended a public event where identifiable as FSW in past 12m	29.9%	35.6%	50.0%	0.67		29.9%	35.0%	0.68
Feels strong sense of unity with FSWs in general		18.7%	16.9%	0.94		17.4%	15.8%	0.94
PHQ-9 raw score (range 0 to 27)	37.0%	9.4	10.7	0.79	36.5%	9.4	10.7	0.80
HIV stigma scale raw score (range 0-9)	9.0%	1.0	0.6	0.38	8.4%	1.0	0.6	0.34
Adapted FSSQ scale raw score (range 0-30)	27.5%	8.6	9.3	0.89	26.1%	8.5	9.3	0.90
Generalized self-efficacy raw score (range 10-40)	34.5%	30.7	32.8	0.67	32.6%	30.8	32.8	0.68

12m: 12 months. FSSQ: Duke-UNC Functional Social Support Questionnaire; FSW: female sex worker; ICC: Intraclass Correlation Coefficient; IQR: inter-quartile range; IVR: interviewer; PHQ-9: Patient Health Questionnaire-9; SES: socio-economic status; SWC: sex work client; ZMW: Zambian Kwacha: 1 Kwacha ~USD 10.

All regressions included study site fixed effects and interviewer random intercepts. Values for male and female IVR are marginal predicted values based on regression coefficients.

[b] ICC is the proportion of all variance in a model without interviewer gender attributable to variation in interviewer identity; not available for Poisson or ordered logistic models. P-value is for a χ^2 test, adjusted for multiple testing across all results shown using the Benjamini-Hochberg method.
